# The effect of alignment uncertainty, substitution models and priors in building and dating the mammal tree of life

**DOI:** 10.1186/s12862-019-1534-9

**Published:** 2019-11-06

**Authors:** Yan Du, Shaoyuan Wu, Scott V. Edwards, Liang Liu

**Affiliations:** 1 Department of Statistics, University of Georgia, 310 Herty Drive, Athens, GA 30606 USA; 2 Jiangsu Key Laboratory of Phylogenomics & Comparative Genomics, School of Life Sciences, Jiangsu Normal University, Xuzhou, Jiangsu 221116 People’s Republic of China; 3 Department of Organismic & Evolutionary Biology, Museum of Comparative Zoology, Harvard University, Cambridge, MA 02138 USA; 4 Liang Liu, Department of Statistics and Institute of Bioinformatics, University of Georgia, 310 Herty Drive, Athens, GA 30606 USA

**Keywords:** Species trees, Gene trees, Divergence times, Phylogenomics, Substitution models, Exons

## Abstract

**Background:**

The flood of genomic data to help build and date the tree of life requires automation at several critical junctures, most importantly during sequence assembly and alignment. It is widely appreciated that automated alignment protocols can yield inaccuracies, but the relative impact of various sources error on phylogenomic analysis is not yet known. This study employs an updated mammal data set of 5162 coding loci sampled from 90 species to evaluate the effects of alignment uncertainty, substitution models, and fossil priors on gene tree, species tree, and divergence time estimation. Additionally, a novel coalescent likelihood ratio test is introduced for comparing competing species trees against a given set of gene trees.

**Results:**

The aligned DNA sequences of 5162 loci from 90 species were trimmed and filtered using trimAL and two filtering protocols. The final dataset contains 4 sets of alignments - before trimming, after trimming, filtered by a recently proposed pipeline, and further filtered by comparing ML gene trees for each locus with the concatenation tree. Our analyses suggest that the average discordance among the coalescent trees is significantly smaller than that among the concatenation trees estimated from the 4 sets of alignments or with different substitution models. There is no significant difference among the divergence times estimated with different substitution models. However, the divergence dates estimated from the alignments after trimming are more recent than those estimated from the alignments before trimming.

**Conclusions:**

Our results highlight that alignment uncertainty of the updated mammal data set and the choice of substitution models have little impact on tree topologies yielded by coalescent methods for species tree estimation, whereas they are more influential on the trees made by concatenation. Given the choice of calibration scheme and clock models, divergence time estimates are robust to the choice of substitution models, but removing alignments deemed problematic by trimming algorithms can lead to more recent dates. Although the fossil prior is important in divergence time estimation, Bayesian estimates of divergence times in this data set are driven primarily by the sequence data.

## Background

There is a growing interest in developing unified models for phylogenomic data analysis that acknowledge a multitude of biological processes. One such model, the multispecies coalescent model, integrates nucleotide substitution processes [1] and the coalescence process [2–6] to deliver estimates of phylogenies that are comprehensive in their acknowledgement of basic biological processes. However, several critical variables, such as sequence alignment uncertainty and the choice of substitution models, have not yet been integrated into these stochastic models, and their effects on phylogenomic inference remain unknown. Alignment uncertainty can be more influential on phylogenetic tree estimation than the specific tree reconstruction methods used [7–10], and its misleading effects depend on the shape of the true phylogeny [11, 12]. A recent study [13] suggested that ‘tiny’ changes in gene sequences could result in changes in estimated phylogenies. Although alignment uncertainty is a critical variable in species tree estimation, the approaches [14, 15] incorporating alignment uncertainty in their overall pipeline of phylogenomic analysis are not yet widely adopted. Moreover, misspecification of substitution models can mislead phylogenetic inference [16–18] and divergence time estimation [19, 20], especially when the substitution model is under-parametrized [21] or rate heterogeneity among sites is ignored [17]. A Bayesian approach [22] and the methods of model adequacy [23] were developed to incorporate and evaluate uncertainty in substitution models in phylogenetic inference. However, most efforts have been devoted to evaluating the effects of alignment uncertainty and substitution models in the context of gene tree estimation; their impacts on species tree inference under the multispecies coalescent have not been fully explored, despite the fact that coalescent species tree estimation appears to be more robust than concatenation methods to taxon sampling, long branch attraction, missing data and other biases [24–26], especially but not exclusively when information content of individual alignments is moderate or high [27, 28].

Placental mammals have received considerable recent attention in phylogenomics, although many nodes are still uncertain (reviewed in [29]) and the models describing their ordinal radiation are still contentious [30–32]. An empirical study suggested a general agreement on paleontological and molecular dates with results consistent with the soft explosive model [33], which were later confirmed and reinforced by Phillips and Fruciano [32] based on simulations and empirical analyses. Liu et al. [34] recently conducted a broad phylogenomic and dating analysis of mammals involving 4388 coding loci. Gatesy and Springer [35] pointed out that some of the alignments in their analysis appeared to be spurious, implying that the study as a whole was compromised. The effect of these alignment errors on the conclusions of [34] seem to be minimal [36], and the analysis of the mammal alignments by Liu et al. [34] is consistent with Phillips’ suggested reconciliation between molecular and paleontological diversification of placental mammals. Another issue not considered by [34] was the effect of prior distributions of divergence times on estimated posterior distributions [37, 38]. Awareness is increasing of the need to test for differences in posterior and prior distributions, so as to gauge whether the signals in the data can overcome the strength of prior distributions [39, 40]. Brown and Smith [41] showed that, for a phylogeny of plants involving 4 genes and 124 taxa, multiple interacting priors could grossly constrain estimated posterior distributions of divergence times, a problem that could potentially affect the mammal data set as well. These exchanges and issues prompted us to explore in a general way the effects of alignment uncertainty and priors on a large phylogenomic data set in mammals, a useful test group with a history of coalescent analyses on large and diverse data sets [42–44]. A larger and improved set of alignments of [34] based on careful codon-based alignment and a state-of-the-art trimming pipeline is now available [45]. The current study comprehensively analyzes this data set of 5162 loci (total alignment length 9,150,597-14,623,557 bp) and 90 species [45] to evaluate the effects of alignment uncertainty, substitution model, and fossil priors on gene tree, species tree, and divergence time estimation in mammals.

## Methods

### Alignments

**Fig. 1:**
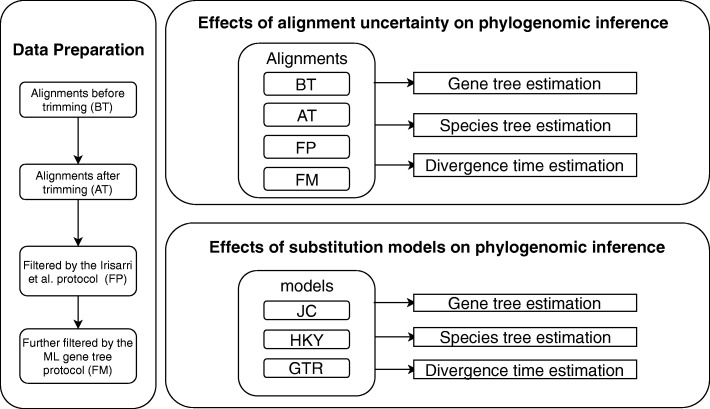
A flow chart of data preparation and analyses conducted in this study. The original mammal alignments were trimmed and filtered, generating 4 sets of alignments - before trimming (BT), after trimming (AT), filtered by the protocol of Irisarri et al. (FP), and further filtered by comparing ML trees with the concatenation tree (FM). In the data analyses, different alignments and substitution models were used to evaluate the effects of alignment uncertainty and the choice of substitution models on phylogenomic inference

The CDS sequences derived from [34] were processed to remove potential low-quality regions and indels. The protein sequences of 5162 loci were aligned by the program Mafft v6 [46], where the protein sequences were translated from the original DNA sequences using EMBOSS [47]. The aligned protein sequences were then back-translated into DNA sequences, retaining the same codons as in the original DNA alignment. The aligned DNA sequences were trimmed by the program trimAl [48] with the option -*gappyout*. The protocol of Irisarri et al. [49], who discussed alignment errors at length, was applied to the trimmed DNA sequences to identify individual sequences within each alignment that appear to generate long-branches due to misalignments or misassemblies. After performing the protocol of Irisarri et al. [49], some qualitatively long branches were still observed in the maximum likelihood (ML) trees; the alignments were therefore further filtered by comparing the ML gene trees with the pruned concatenation tree. For each gene, the sequences corresponding to any unusually long terminal branches in the ML gene tree (those branches 5X or longer than the corresponding branches of the pruned concatenation tree) were removed. Finally, the first and second codon positions (C12) were extracted from 4 sets of alignments - before trimming (BT), after trimming (AT), filtered by the protocol of Irisarri et al. (FP), and further filtered by comparing ML trees with the concatenation tree (FM) (Fig. 1). In our analysis, the extracted C12 positions of the BT, AT, FP, and FM alignments were used to assess uncertainty of alignments and its effect on gene trees, species trees, and divergence time estimation (Fig. 1). The final CDS and C12 alignments were deposited in figshare (https://figshare.com/articles/cds_5162.zip/6031190; [45]).

Before-trimming, the BT alignments of 5162 loci for 90 species had a total of 14,623,557 base pairs. All 5162 loci include at least 80 sequences, with an average of 86.24 sequences per locus. Using trimAl, 38% of sites were trimmed from the alignments to produce the AT alignments. The protocol of Irisarri et al. [49] removed 13,181 sequences, i.e., 3% of a total of 445,209 sequences of the AT alignments, to produce the FP alignments. The FP alignments were further filtered by the ML protocol and an additional 392 sequences were removed to produce the FM alignments. The extracted C12 positions of the BT, AT, FP, and FM alignments were summarized with respect to the proportion of informative sites, GC contents, and base frequencies (Additional file 1: Figure S1). The average proportion of informative sites is 30, 43, 41, 41% for the BT, AT, FP, and FM alignments, respectively (Additional file 1: Figure S1a). The *χ*^2^ test identifies 88% of genes whose proportion of informative sites of the AT alignments is significantly higher than that of the BT alignments (Additional file 1: Figure S1b). In contrast, the proportion of significant genes is 14% and only 0.5% for AT versus FP and FP versus FM, respectively (Additional file 1: Figure S1b). Both the average proportion of informative sites and the *χ*^2^ test indicate that trimming can significantly increase the proportion of informative sites, but the protocol of Irisarri et al. [49] and the ML protocol have little impact on the proportion of informative sites. The analysis for GC contents and base frequencies shows no significant difference among the BT, AT, FP, and FM alignments (Additional file 1: Figure S1c-d); the proportion of AT nucleotides (52%) is consistently greater than the proportion of GC nucleotides (48%) in all alignments (Additional file 1: Figure S1d).

### Gene tree estimation

To evaluate the effect of alignment uncertainty on gene tree estimation, maximum likelihood (ML) and bootstrap gene trees were built from the BT, AT, FP, and FM alignments using RAxML v8.2.11 [50] with the GTRCAT model. The RAxML command line for building the ML and 100 bootstrap gene trees is raxmlHPC-AVX -f a -#100 -s inputfile -n outputfile -m GTRCAT -p randomseed1 -x randomseed2. We chose the GTRCAT model because it substantially reduced the computational time of the ML and bootstrap analyses in RAxML. The ML gene trees of the AT, FT, and FM alignments were compared with those generated from the BT alignments using the normalized Robinson Foulds distance (NRFD, i.e., the Robinson Foulds distance divided by the maximum possible distance) [51]. For genes with missing species, the NRFD was calculated by pruning two gene trees to a set of species that appear in both genes. Let $D\left(M{L}_i^m,M{L}_i^{BT}\right)$ be the distance between the $M{L}_i^m$ tree of the alignment *m* = {*AT*, *FP*, *FM*} and the $M{L}_i^{BT}$ tree of the BT alignment for gene *i*. For each gene, the 99% quantile of the tree distances was calculated using the ML and 100 bootstrap gene trees estimated from the BT alignments. The distance $D\left(M{L}_i^m,M{L}_i^{BT}\right)$ between two ML gene trees is then standardized by the 99% quantile of the tree distances. If the standardized tree distance is greater than 1, the distance between two ML gene trees falls outside the 99% confidence interval of the tree distances, indicating that the ML tree of alignment *m* = {*AT*, *FP*, *FM*} is significantly incongruent with the ML tree of the BT alignment. However, if a tree distance $D\left(M{L}_i^m,M{L}_i^{BT}\right)$ falls in the 99% confidence interval of the tree distances, it is not necessarily true that the corresponding ML tree of the *m* = {*AT*, *FP*, *FM*} alignment is in the 99% confidence interval of the trees around the ML tree of the BT alignments. Moreover, we calculate the average bootstrap percentage for each gene tree and then fitted a linear regression line for the average bootstrap percentages for AT versus BT, FP versus BT, and FM versus BT, respectively, to explore the effect of alignment on estimates of bootstrap support. The paired *t* test was utilized to find significance for the logarithm of average bootstrap percentages of the BT, AT, FP, and FM alignments. The linear regression and paired *t* analyses were performed in R using functions *lm*() and *t.test*(). To evaluate the effect of substitution models on gene tree estimation, ML and bootstrap gene trees were built by RAxML with the JC, K80, and GTR models for the FM alignments. For each gene, the NRFD between the K80 (or GTR) gene tree and the JC gene tree was calculated, and then was standardized by the 99% quantile of the tree distances as described above, where the 99% quantile of the tree distances was calculated from the ML and bootstrap gene trees estimated with the JC model. We again fitted a linear regression line for the average bootstrap percentages of K80 versus JC and GTR versus JC, and we conducted the paired *t* test to find significance for the logarithm of average bootstrap percentages estimated with the JC, K80, GTR models.

### Species tree reconstruction

To evaluate the effect of alignment uncertainty on species tree estimation, species trees were estimated from the bootstrap gene trees of the BT, AT, FP, FM alignments using the species tree estimation methods ASTRAL [52], NJst [53], and STAR [54]. Bootstrap gene trees were obtained using RAxML with the GTRCAT model. The ASTRAL, NJst, and STAR trees were summarized by a majority rule consensus tree, respectively. In each case, the consensus tree was used as the estimate of the species tree. The NJst and STAR methods were implemented in the R package Phybase v2.0 [55], whereas the ASTRAL trees were reconstructed by the command line *java -jar astral.5.5.6.jar -i input -o output*. The bootstrap concatenation analysis was conducted with 100 bootstrap replicates for the BT, AT, FP, FM alignments using RAxML with the GTRCAT model. To evaluate the effects of substitution models on species tree estimation, species trees were reconstructed from the gene trees estimated with the JC, K80, and GTR models for the FM alignments. The ASTRAL, STAR, NJst trees were reconstructed from bootstrap gene trees and summarized by a majority rule consensus tree (see above for detailed description). In the concatenation analysis, 100 bootstrap concatenation trees were built from the alignments concatenated across 5162 loci by RAxML with the JC, K80, and GTR model.

We calculated the Robinson-Foulds distance [56] between the reconstructed species trees from various alignments or substitution models. A coalescent bootstrap likelihood ratio test (cbLRT) [57] was introduced to identify significant incongruence among the concatenation, ASTRAL, NJst, and STAR trees given a set of estimated gene trees. Thus far, the strength of the data behind a given coalescent species tree has most often been gauged using the bootstrap, which is only an indirect method of comparing two species trees in a coalescent context. Given a collection of gene trees known without error, two species trees T_0_ (the null tree) and T_1_ (the alternative tree with a higher likelihood score) are evaluated by the LRT statistic *t* = 2(log(*L*_1_) − log(*L*_0_)), where *L*_0_ and *L*_1_ are the likelihoods of T_0_ and T_1_ calculated by the species tree estimation program MP-EST [58]. The null distribution of the test statistic *t* is approximated by a parametric bootstrap technique. Specifically, *n* bootstrap samples of gene trees are generated from the null tree T_0_ with branch lengths estimated by MP-EST. The null distribution of the test statistic *t* is approximated by the test statistics {*t*_1_, …, *t*_*n*_} calculated from *n* bootstrap samples, and $pvalue=P\left(t\ge {t}^{\ast }|{T}_0\right)\approx \frac{\# of\ {t}_i\ge {t}^{\ast }}{n}$, where t^*^ is the test statistic calculated from the real data. If *pvalue* is less than or equal to a prespecified *α* (typically, *α* = 0.05), we conclude that incongruence between two trees T_0_ and T_1_ is significant and the LRT favors the alternative tree T_1_. For gene trees estimated from the BT alignments, the concatenation, ASTRAL, NJst, and STAR species trees were sorted by their loglikelihoods, and then we performed the LRT to compare two consecutive trees. Similarly, the LRT was conducted for the concatenation, ASTRAL, NJst, and STAR trees estimated from the AT, FP, and FM alignments. Since the loglikelihood is calculated under the multispecies coalescent model, the cbLRT is in favor of the trees (ASTRAL, NJst, STAR trees) estimated from the coalescent methods.

### Divergence time analysis

Divergence times were fit to the ASTRAL, STAR, and concatenation trees using MCMCTREE [59, 60]. This study adopted the same 21 fossil calibration points in Liu et al. [34], which have a relatively even distribution across the tree (see Fig. S7 in Liu et al. [34]). The ASTRAL, STAR, and concatenation trees were estimated from extracted C12 positions of the FM alignment. The C12 NJst tree was identical to the C12 STAR tree produced here and to the C12 STAR tree in [34]. Divergence times were also fit to the concatenation tree in [34] in order to compare the time estimates produced from the new alignments (5162 loci) in this study and those produced from the alignments (4388 loci) in [34]. Thus, divergence times were fit to 4 species trees – ASTRAL, STAR, concatenation4388, concatenation5162. Liu et al. [34] retained 3867 genes for dating analysis and divided them into five quintiles. Two hundred genes were randomly selected from the gene group quintile 1 of 773 genes. Divergence time estimation was based on the selected 200 genes of the BT, AT, FP, and FM alignments. We used the same parameter settings for the MCMCTREE analysis as those in [34]. Specifically, the number of partitions was set to 200 and the clock model was set to independent rates model. To evaluate the effect of alignment uncertainty on divergence time estimation, the divergence times estimated from the AT, FP, and FM alignments were compared with those of the BT alignments for the ASTRAL, STAR, concat4388, and concat5162 trees. To evaluate the effect of substitution models, divergence times were fit to the STAR tree estimated from the FM alignments using MCMCTREE with the JC, K80, and HKY models. Finally, we evaluated the effect of the fossil prior on divergence time estimation by running MCMCTREE without sequence data. The divergence times produced from the fossil prior were compared with the posterior distribution of divergence times fitted to the STAR tree given the FM alignments. The divergence times estimated from the FM alignments for the STAR and concatenation4388 trees were also compared with those in [34]. We compared two posterior distributions of divergence times based on the standardized divergence times defined as follows. Let *x* = {*x*_1_, …, *x*_81_} and *y* = {*y*_1_, …, *y*_81_} be the Bayesian estimates (i.e., posterior means) of the divergence times at 81 internodes of the species tree (excluding 8 non-mammal outgroup species) for the BT and AT alignments. Suppose that the 95% posterior interval of the divergence time *x*_*i*_ is (*x*_*i*_ − *d*_*i*_, *x*_*i*_ + *d*_*i*_). The standardized divergence time is defined as ${y}_i^{\ast }=\left({y}_i-{x}_i\right)/{d}_i$. Thus, a standardized divergence time ${y}_i^{\ast }>1$ indicates that the divergence time *y*_*i*_ falls outside the 95% posterior interval of *x*_*i*_, and we conclude that divergence time *y*_*i*_ estimated from the AT alignments is significantly different from the divergence time *x*_*i*_ estimated from the BT alignments.

## Results

### Gene tree estimation

The distance analysis identified only 38 (0.7%), 43 (0.8%), and 41 (0.8%) gene trees with standardized tree distance >1 for the AT, FP, and FM alignments, respectively (Additional file 1: Figure S2a). Thus, the distance analysis does not find significant difference for most gene trees estimated from the BT, AT, FP, and FM alignments. In the linear regression analysis for the average bootstrap percentages of different alignments, the slope of the linear regression line for AT versus BT, FP versus BT, and FM versus BT is $\hat{\beta}$ = 1.008, 0.988, 0.999, respectively (Additional file 1: Figure S2b). Since the slopes are close to 1, the average bootstrap percentages of the BT, AT, FP, and FM gene trees are consistent across alignments for each of the 5162 loci. Moreover, the paired *t* test finds no significant difference among the average bootstrap percentages of the BT, AT, FP, and FM gene trees. The tree distance analysis also suggests that there is no significant difference among the gene trees estimated with the JC, K80, and GTR models, because the standardized tree distance is less than 1 for the JC versus K80 and JC versus GTR comparisons (Additional file 1: Figure S2a). The linear regression analysis suggests that the average bootstrap percentages estimated with the JC, K80, and GTR models are consistent across 5162 loci (Additional file 1: Figure S2b). The slope of the linear regression line for K80 versus JC and GTR versus JC is $\hat{\beta}$ = 1.008, 1.006, respectively. The average bootstrap percentages for substitution models are more concentrated around the fitted regression line than those for alignment uncertainty (Additional file 1: Figure S2b). The paired *t* test finds no significant difference among the average bootstrap percentages estimated for the JC, K80, GTR substitution models.

### Species tree estimation

**Fig. 2:**
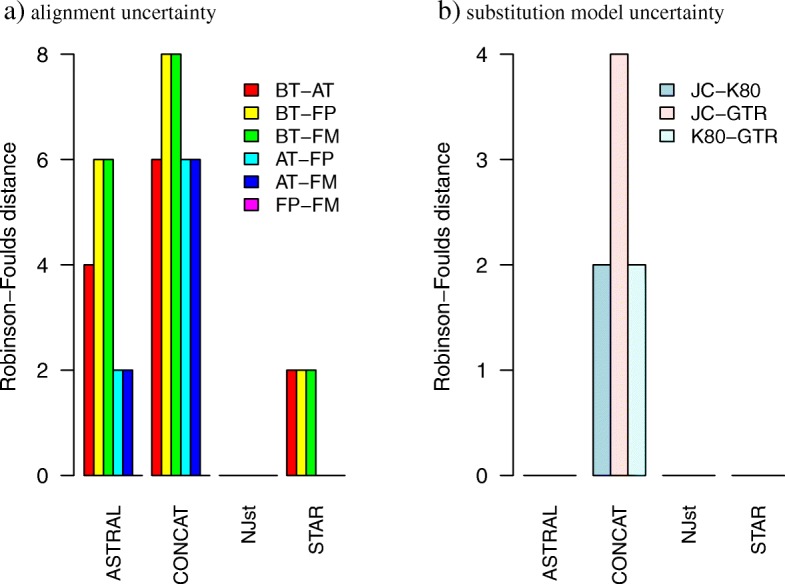
The effects of alignment uncertainty and the choice of substitution models on species tree estimation. **a** The pairwise distances of the ASTRAL, concatenation, NJst, and STAR trees estimated from the BT, AT, FP, and FM alignments. **b** The pairwise distances of the ASTRAL, concatenation, NJst, and STAR trees estimated with the JC, K80, and GTR models. Note that the pairwise distances of the ASTRAL, NJst, and STAR trees are equal to 0

The average Robinson-Foulds distance among the concatenation trees estimated from the BT, AT, FP, and FM alignments is 5.7 (Fig. 2a). By contrast, the average distance among the ASTRAL, STAR and NJst trees is 3.3, 1 and 0, respectively (Fig. 2a). Among the 6 comparisons (BT-AT, BT-FP, BT-FM, AT-FP, AT-FM, FP-FM), the BT versus FM comparison has the largest average Robinson-Foulds distance for all 4 species tree estimation methods (ASTRAL, concatenation, NJst, STAR), and the average distance decreases for the BT-FP and BT-AT comparisons (Fig. 2a). This decreasing trend in tree distance is expected because the BT and FM alignments are the two ends of the trimming and filtering process and they are the most dissimilar alignments compared to the other two intermediate products (AT and FP alignments) of the trimming and filtering process. The average tree distances for the BT-AT, BT-FP, BT-FM comparisons are larger than the average distances for the AT-FP, AT-FM, FP-FM comparisons, and the average tree distance is 0 for the FP-FM comparison for all 4 species tree reconstruction methods (Fig. 2a). Thus, trimming is the major factor of alignment uncertainty influencing species tree estimation, whereas our ML protocol of filtering additional long branches of the ML gene trees has little impact on species tree estimation. The average Robinson-Foulds distance of the concatenation trees estimated with the JC, K80, and GTR models is 2.7, whereas the average distance is 0 for the ATRAL, STAR, NJst trees (Fig. 2b), indicating that the coalescent methods for estimating species trees are more robust to the choice of substitution models than the concatenation methods. In the cbLRT, the concatenation tree has the smallest loglikelihood and the NJst tree has the largest loglikelihood for all alignments (Additional file 1: Figure S3a-d) and substitution models (Additional file 1: Figure S3e-f). The cbLRT consistently rejects the concatenation tree when compared to the ASTRAL tree (*pvalue* < 0.01), and further favors the NJst tree over the ASTRAL tree (*pvalue* < 0.01) across all alignments and substitution models (Additional file 1: Figure S3a-f). Thus, incongruence among the concatenation, ASTRAL, and NJst trees is statistically significant, and the NJst tree is the best tree selected by the cbLRT. The NJst tree with high bootstrap support values (> 90) is topologically identical with the species tree in Liu et al. (see Fig. 1 in [34]).

### Divergence time estimation

**Fig. 3:**
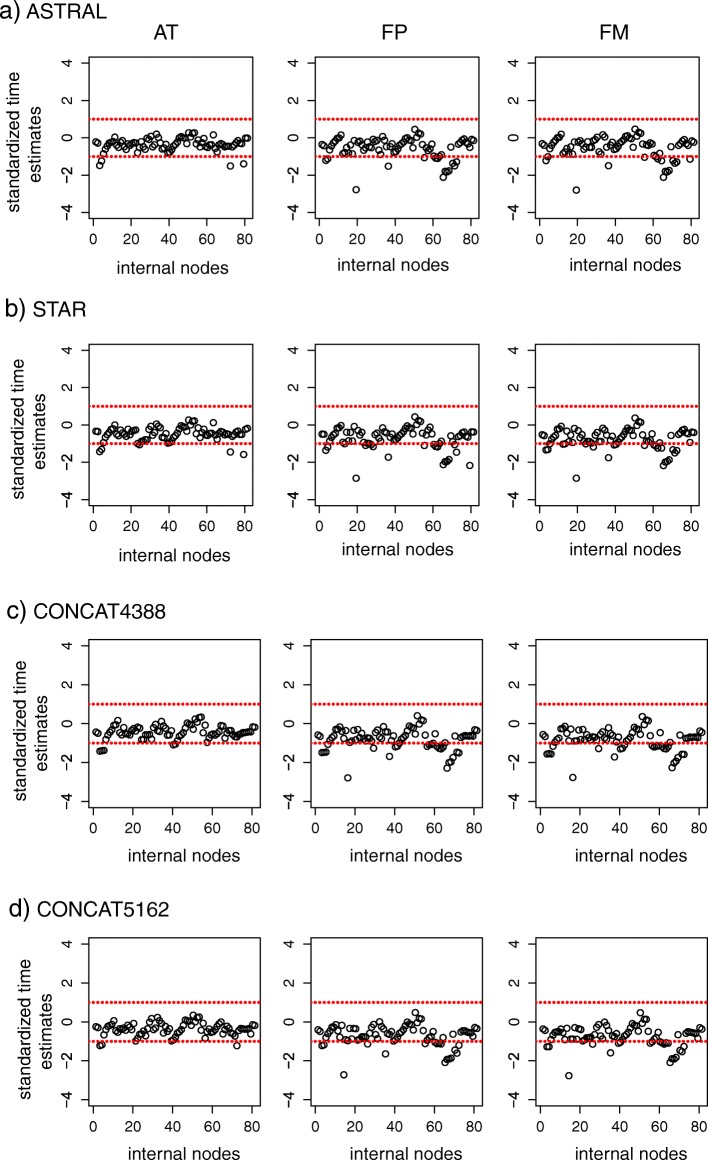
The scatter plot of the standardized time estimates for the BT, AT, FP, FM alignments. The Bayesian estimates of divergence times for the AT, FP, and FM alignments are standardized by subtracting the Bayesian estimates for the BT alignments and then divided by the half length of the 95% posterior interval for the BT alignments. The standardized time estimates are plotted for **a**) the ASTRAL tree, **b**) the STAR tree, **c**) the concatenation tree in Liu et al. [34], and d) the concatenation tree estimated from the FM alignments of the C12 data sets in this study

**Fig. 4:**
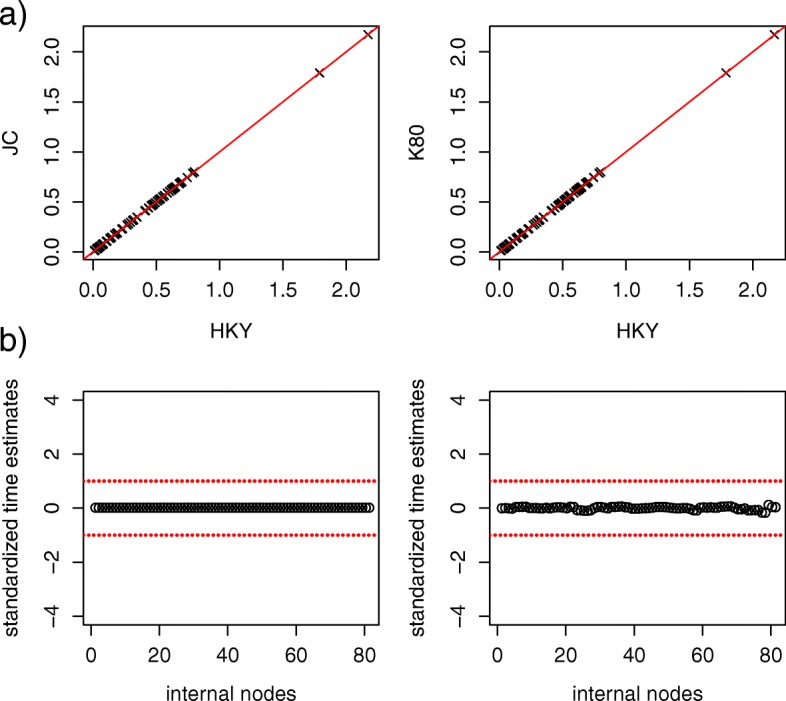
The effect of the choice of substitution models on divergence time estimation. **a** The scatter plot of the time estimates from the JC model versus the HKY model (left panel), and the K80 model versus the HKY model. The straight line is the 1:1 line. **b** The scatter plot of standardized time estimates. The Bayesian estimates of divergence times for the JC and K80 models are standardized by subtracting the Bayesian estimates for the HKY model and then divided by the half length of the 95% posterior interval for the HKY model. The points outside the two dashed lines indicate that the corresponding time estimates from the JC model (left panel) or from the K80 model (the right panel) are significantly different from the time estimates from the HKY model

The points in the scatter plot of the divergence times estimated from the AT, FP, and FM alignments against those from the BT alignments are close to the 1:1 line (Additional file 1: Figure S4). High similarity of divergence time estimates across different alignments is consistently exhibited for all 4 species trees (Additional file 1: Figure S4). In addition, most standardized time estimates of the 4 species trees for the AT alignments fall in the interval [− 1, 1] (Fig. 3), indicating that trimming does not have major effect on divergence time estimation. In contrast, an increasing number of points for the FP and FM alignments fall below the − 1 line (Fig. 3), indicating that removing sequences (due to the protocol of Irisarri et al.) may result in more recent time estimates for some internodes. Nevertheless, two diversification rate shifts at 54 Ma and 83 Ma (Additional file 1: Figure S5) estimated by the birth-death-shift model [61] are consistent with the estimates in Liu et al. [34]. The choice of the substitution model appears to have little effect on divergence time estimation, because the points in the scatter plots are close to the 1:1 line (Fig. 4a) and all standardized divergence times of the JC and K80 models are between − 1 and 1 when compared to the divergence times of the HKY model (Fig. 4b).

**Fig. 5:**
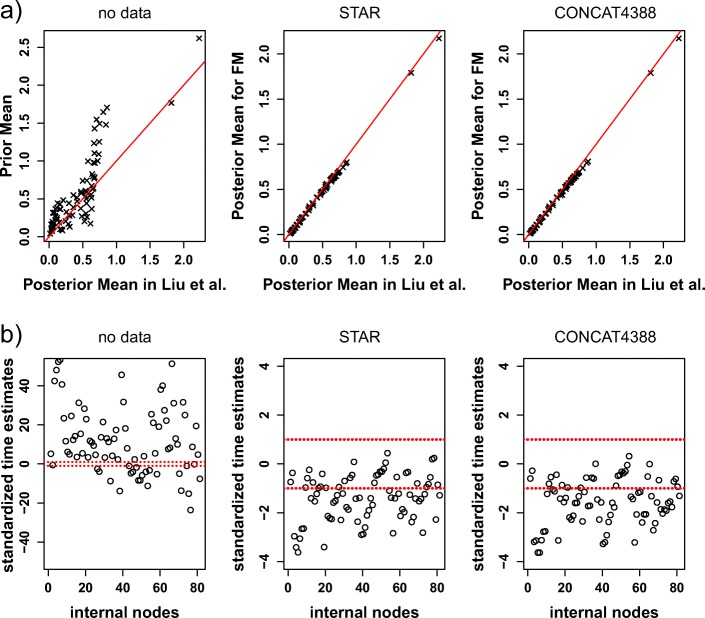
Comparison of the time estimates of the fossil prior and the time estimates for the new alignments in this study with the time estimates in Liu et al. [34]. **a** The scatter plot of the prior mean versus the posterior mean (left panel), the posterior mean for the new alignments (FM) in this study versus the posterior mean in [34] fitted to the STAR tree (middle panel), and the posterior mean for the new alignments (FM) in this study versus the posterior mean in [34] fitted to the concatenation tree (right panel). The straight line is the 1:1 line. **b** The standardized time estimates. The points outside the two dashed lines indicate that the corresponding time estimates from the prior (or from the new alignments) are significantly different from the time estimates in [34]

The prior mean of divergence times tends to be larger than the corresponding posterior mean, because there are more points above the 1:1 line (Fig. 5a). Meanwhile, 98% standardized time estimates from the prior distribution without the sequence data are either less than − 1 or greater than 1 (Fig. 5b), suggesting that there is substantial difference between the posterior and prior distributions of divergence times. Thus, although the fossil prior is important in divergence time estimation, the Bayesian estimates of divergence times are primarily driven by the sequence data. In contrast, the estimated divergence times from the new alignments versus the alignments in [34] are close to the 1:1 line (Fig. 5a), but because the posterior standard deviations of those points are very small, there is still a large proportion (59% for STAR and 68% for concat4388) of the standardized time estimates falling outside the interval [− 1, 1] (Fig. 5b). Interestingly, all points outside the interval [− 1, 1] fall below the − 1 line, indicating that the new alignments in this study tend to produce more recent time estimates, in contrast to the time estimates in Liu et al. [34].

## Discussion

Phylogenetic inference may be compromised by several types of errors, some of which can be reduced with further sampling but all of which necessarily incur a tradeoff between bias and variance [62]. Estimation errors of phylogenies and divergence times reflect the degree of uncertainty in estimating phylogenetic parameters due to random sampling of genes and taxa [63] . Large estimation errors result in unresolved phylogenies and wide confidence intervals (or Bayesian credible regions) for divergence time estimates but can be reduced by increasing the sample size of genes or alignment lengths. In contrast, systematic errors due to data artifacts and model misspecifications cannot be alleviated by sampling more data; a large sample size often leads to even more pronounced systematic errors and can further bias phylogenetic inference. For the mammal data set, alignment uncertainty and substitution model misspecification have little impact on gene tree estimation. Moreover, the gene-tree-based species tree estimation methods (ASTRAL, STAR, NJst) are robust to alignment uncertainty, because a small number of bad gene trees will not bias the species tree estimates of those methods. In contrast, the concatenation methods are sensitive to alignment uncertainty of the mammal data set, because a sufficiently misleading locus could potentially dominate the estimate of the species tree, which is more likely when most loci have few informative sites and one locus (perhaps due to very long branches) has many more informative sites than average. Similarly, the choice of substitution models of the mammal data set has a greater effect on the concatenation approach than on the gene-tree-based species tree estimation methods tested here. It should be noted, however, that more variable performance of concatenation compared to coalescence methods given the different alignments does not necessarily signify higher accuracy.

In our analyses, alignment uncertainty had a strong influence on divergence time estimation for the mammal data set. Removing problematic sites (trimming) and sequences (filtering) may yield more recent dates at some internal nodes of the species tree. This effect of alignment uncertainty is primarily due to sequence filtering, whereas the trimming and the choice of substitution models had little effect on divergence time estimation. In addition to alignment uncertainty, other factors including the choice of calibration scheme and clock models (i.e., autocorrelated or independent rates model) may also have a strong impact on divergence dates [64–66]. Moreover, trimming rather than filtering is the main alignment factor influencing the species tree topology. Trimming is not universally agreed to be a positive step in improving alignment quality. Tan et al. [67] suggested that trimming worsened phylogenetic analysis in many cases. Edwards et al. [68] suggested that their analyses showed only slight reductions in phylogenetic accuracy under unusually severe levels of trimming (e.g., > 40% of sites trimmed). In our analyses, the sequences were trimmed on average by about 38%. The subsequent phylogenetic analysis showed that the reconstructed gene trees were not significantly impacted by trimming. Our addition of a further filtering step on top of those proposed by Irisarri et al. [49] is easy to compute and may prove useful in automated pipelines in some contexts, but with the mammal data analyzed here it had minimal impacts. Overall our results support the tree topology of Liu et al. [34] and others, adding another analysis favoring the Atlantogenata hypothesis [44].

As for the species tree approaches, the coalescent models were less likely to be influenced by trimming or substitution models than the concatenation methods. Trimming and substitution models worsen concatenated analyses probably because inaccuracies in the alignment or substitution model assumed for each gene compounds a misspecified model. In contrast, individual alignments, which do not have much information, would not be as strongly affected by model misspecifications, resulting in a more consistent species tree. The low information content of individual alignments or genes has been flagged as a criticism of coalescent methods, but in some cases this dearth of information for each gene may be an advantage when models are misspecified. Esselstyn et al. [44] achieved robust estimation of the tree for placental mammals despite the fact that many of their genes did not recover specific uncontested branches in the species tree. We did not attempt another promising type of filtering, namely enriching for genes that recover an uncontested branch in the species tree [69]. Shen et al. [13] showed that single rogue genes, or sites within genes, could have a substantial effect on phylogenomic analyses conducted under concatenation, but less so for species tree methods, which they did not test extensively. Their study is not completely comparable to ours; whereas they focused primarily on sites which strongly supported one tree or another (using alignments that were fixed and presumably accurate), we focused on alignment uncertainty. Still, the sensitivity to inclusion of individual sites in their study was similar to changes in alignment of our study. We suggest that by removing a specific site (random or otherwise) from every gene in a phylogenomic study for coalescent methods, but not for concatenation methods (where they tended to remove individual sites or genes from the entire alignment), the results in their Fig. 4 do not allow a clear comparison of the coalescent and concatenation methods. To the extent that our trimming analyses removed sites considered random in their study, without strong support for alternative trees, our results are similar to theirs in showing very little effect on phylogenetic analysis using coalescent methods. This study suggests that coalescent-based methods are insensitive to alignment uncertainty. Other two-stage methods (first estimating gene trees and then estimating species trees from the estimated gene trees) would get similar insensitivity to alignment even though they are not coalescent-based methods, especially when those methods only use gene tree topologies to infer species trees. However, the coalescent-based methods (maximum tree [70] or STEM [71]) that use gene tree branch lengths in estimating species trees may have more sensitivity to alignment, because changing the alignment will change the estimated branch lengths in the gene trees. It would also be interesting to compare the Bayesian multispecies coalescent models (BEST [72] or *BEAST) and evaluate their sensitivity to alignment uncertainty, although such analyses are beyond our available computational resources. Nevertheless, we would expect that BEST and *BEAST are more sensitive to alignment than two-stage topology approaches but less sensitive than concatenation.

Divergence times were estimated under the concatenation model in MCMCTREE. This approach does not take into account the effect of coalescence process, where we expect gene coalescence times to pre-date species divergence times, sometimes substantially [73, 74]. Angelis and dos Reis [75] suggested that estimates of divergence times could be substantially impacted by ancestral population size and incomplete lineage sorting. However, currently available dating methods incorporating the multi-species coalescent model are computationally expensive and impractical for big datasets [75]. There is still a lingering disconnect between coalescent methods for phylogenetic topology and compatible methods for estimating divergence times. Still, we were able to demonstrate that there is substantial difference between the fossil prior and the posterior distribution of divergence times, indicating that our Bayesian estimates of divergence times are driven by the sequence data rather than the pre-specified fossil priors [39].

The coalescent bootstrap likelihood ratio test [57] that we used to assesses the fit of various species tree estimation methods to a given gene tree data set should be helpful in allowing phylogeneticists to compare different trees directly to one another via a LRT. The test offers a convenient way to compare the strength of a set of gene trees to reject alternative species trees. Such tests are preferred over bootstrap measures of support, which have been widely used in the context of coalescent species trees [76, 77], but which are only indirect ways to test competing phylogenetic hypotheses. The test also highlights a key difference between popular methods for species tree estimation, such as ASTRAL, which perform well but are not based on an explicit likelihood model, and methods like MP-EST, *BEAST or BPP, which are founded, to varying degrees of complexity, on the full multispecies coalescent likelihood model [58, 78, 79]. However, the test described here is limited because it assumes that the gene trees are given without error. This is clearly a drawback and might result in rejecting a proposed species tree unnecessarily – a Type I error. In the case of ‘two-step’ species tree methods, gene trees are estimated and should not be taken as given. Our proposed test can be improved by generating bootstrap samples of DNA sequences using the estimated gene trees to produce sets of bootstrapped gene trees and thus achieve a more reliable likelihood of species trees. Full Bayesian comparison of topologies in a coalescent framework are also possible using Bayes Factors [2], although such tests are difficult to implement on large data sets such as we have analyzed here.

This study analyzed the alignments of 90 mammal species to evaluate the effects of alignment artifacts and substitution model misspecification on phylogenetic inference. Although mammals represent a major group of the tree of life, a general conclusion on the effects of alignment and substitution model artifacts requires phylogenetic analyses of genome-scale sequence data for the species sampled from other parts of the Tree of Life. Simulation will certainly also be useful for encompassing a wide range of biological scenarios under which phylogenomic data may be generated.

## Conclusions

In this study, the aligned DNA sequences of 5162 loci from 90 species were trimmed and filtered using trimAL and two filtering protocols. The final dataset containing 4 sets of alignments was used to evaluate the effects of alignment uncertainty, substitution models, and fossil priors on gene tree, species tree, and divergence time estimation. Our analyses suggest that alignment uncertainty of the mammal data set and the choice of substitution models have little impact on tree topologies yielded by coalescent methods for species tree estimation, whereas they are more influential on the concatenation trees. Given the choice of calibration scheme and clock models, divergence time estimates are robust to the choice of substitution models, but removing alignments deemed problematic by trimming algorithms can lead to more recent dates. Although the fossil prior is important in divergence time estimation, Bayesian estimates of divergence times in this data set are driven primarily by the mammal sequence data.

## Supplementary information

**Additional file 1: Figure S1.** Summary of the BT, AT, FP, and FM alignments. a) Boxplot of the proportion of informative sites across 5162 loci. b) The proportion of significant genes for which the number of informative sites is significantly different between two alignments. c) Boxplot of GC content across each of 5162 loci of the BT, AT, FP, and FM alignments. d) The bar plot of base frequencies for each of the four alignments. **Figure S2.** The effect of alignment and substitution model uncertainty on gene tree estimation. ML and bootstrap gene trees were estimated from the BT, AT, FP, and FM alignments. a) Histogram of the standardized tree distance across 5162 loci. The distance between two ML gene trees is standardized by the maximum distance calculated by bootstrap gene trees. In the first three plots, the ML gene trees of the AT, FP, and FM alignments are compared with the ML gene trees of the BT alignments, and the maximum distance is calculated by the bootstrap gene trees of the BT alignments. In the last two plots, the ML gene trees estimated with the K80 and GTR models are compared with the ML gene trees estimated with the JC model, and the maximum distance is calculated by the bootstrap gene trees estimated with the JC model. b) Scatter plot of bootstrap percentages across 5162 loci. The first three plots are the bootstrap percentages of the gene trees estimated from the AT, FP, and FM alignments against the BT alignments, and the last two plots are the bootstrap percentages of the gene trees estimated with the K80 and GTR models against the JC model. **Figure S3.** Significant incongruence among the estimated species trees by a likelihood ratio test. The LRT is conducted to compare the concatenation tree (the null tree) versus the ASTRAL tree (the alternative tree), and the ASTRAL tree (the null tree) versus the NJst tree (the alternative tree) for a) BT, b) AT, c) FP, d) FM alignments and e) JC, f) K80 substitution models. The log likelihoods of three trees are calculated by MP-EST, and then subtracted from the minimum of three loglikelihoods. Asterisks indicate that the test rejects the null tree and favors the alternative tree with *pvalue* < 0.01. **Figure S4.** The effect of alignment uncertainty on divergence time estimation. The posterior means of divergence times estimated with MCMCTREE for the AT, FP, and FM alignments are plotted against the posterior means of divergence times for the BT alignments for a) the ASTRAL tree, b) the STAR tree, c) the concatenation tree in Liu et al. [34], and d) the concatenation tree estimated from the FM alignments of the C12 data sets in this study.

## Data Availability

The datasets supporting the conclusions of this article are available in the figshare repository, https://figshare.com/s/142b6f98bc9c13a0bd7f [80].

## Abbreviations

AT: After trimming
BT: Before trimming
CDS: Coding sequences
FM: Filtered by comparing ML trees with the concatenation tree
FP: Filtered by the protocol of Irisarri et al.
GTR: General time reversible model
JC: Jukes-Cantor model
K80: Kimura 1980 model
ML: Maximum likelihood
