# Supplementary material for: The effect of alignment uncertainty, substitution models and priors in building and dating the mammal tree of life
Source: BMC Evol Biol. 2019 Nov 6;19:203. doi: 10.1186/s12862-019-1534-9 (PMC6833305; doi:10.1186/s12862-019-1534-9)
Supplement: Supplementary file 1 — Additional file 1: Figure S1. Summary of the BT, AT, FP, and FM alignments. a) Boxplot of the proportion of informative sites across 5162 loci. b) The proportion of significant genes for which the number of informative sites is significantly different between two alignments. c) Boxplot of GC content across each of 5162 loci of the BT, AT, FP, and FM alignments. d) The bar plot of base frequencies for each of the four alignments. Figure S2. The effect of alignment and substitution model uncertainty on gene tree estimation. ML and bootstrap gene trees were estimated from the BT, AT, FP, and FM alignments. a) Histogram of the standardized tree distance across 5162 loci. The distance between two ML gene trees is standardized by the maximum distance calculated by bootstrap gene trees. In the first three plots, the ML gene trees of the AT, FP, and FM alignments are compared with the ML gene trees of the BT alignments, and the maximum distance is calculated by the bootstrap gene trees of the BT alignments. In the last two plots, the ML gene trees estimated with the K80 and GTR models are compared with the ML gene trees estimated with the JC model, and the maximum distance is calculated by the bootstrap gene trees estimated with the JC model. b) Scatter plot of bootstrap percentages across 5162 loci. The first three plots are the bootstrap percentages of the gene trees estimated from the AT, FP, and FM alignments against the BT alignments, and the last two plots are the bootstrap percentages of the gene trees estimated with the K80 and GTR models against the JC model. Figure S3. Significant incongruence among the estimated species trees by a likelihood ratio test. The LRT is conducted to compare the concatenation tree (the null tree) versus the ASTRAL tree (the alternative tree), and the ASTRAL tree (the null tree) versus the NJst tree (the alternative tree) for a) BT, b) AT, c) FP, d) FM alignments and e) JC, f) K80 substitution models. The log likelihoods of three trees a [file 12862_2019_1534_MOESM1_ESM.docx]

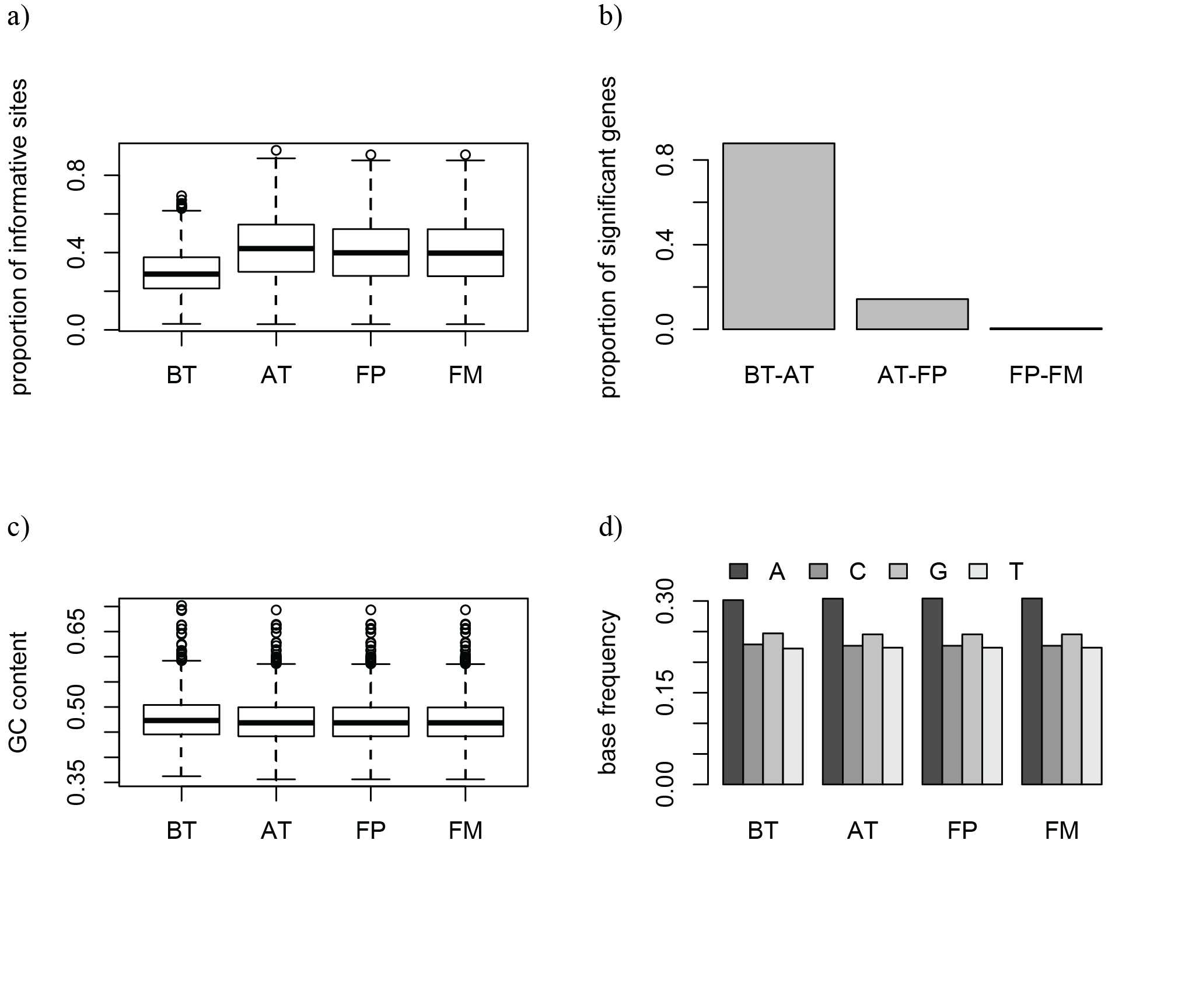


Figure S1. Summary of the BT, AT, FP, and FM alignments. a) Boxplot of the proportion of informative sites across 5,162 loci. b) The proportion of significant genes for which the number of informative sites is significantly different between two alignments. c) Boxplot of GC content across each of 5,162 loci of the BT, AT, FP, and FM alignments. d) The bar plot of base frequencies for each of the four alignments.

**
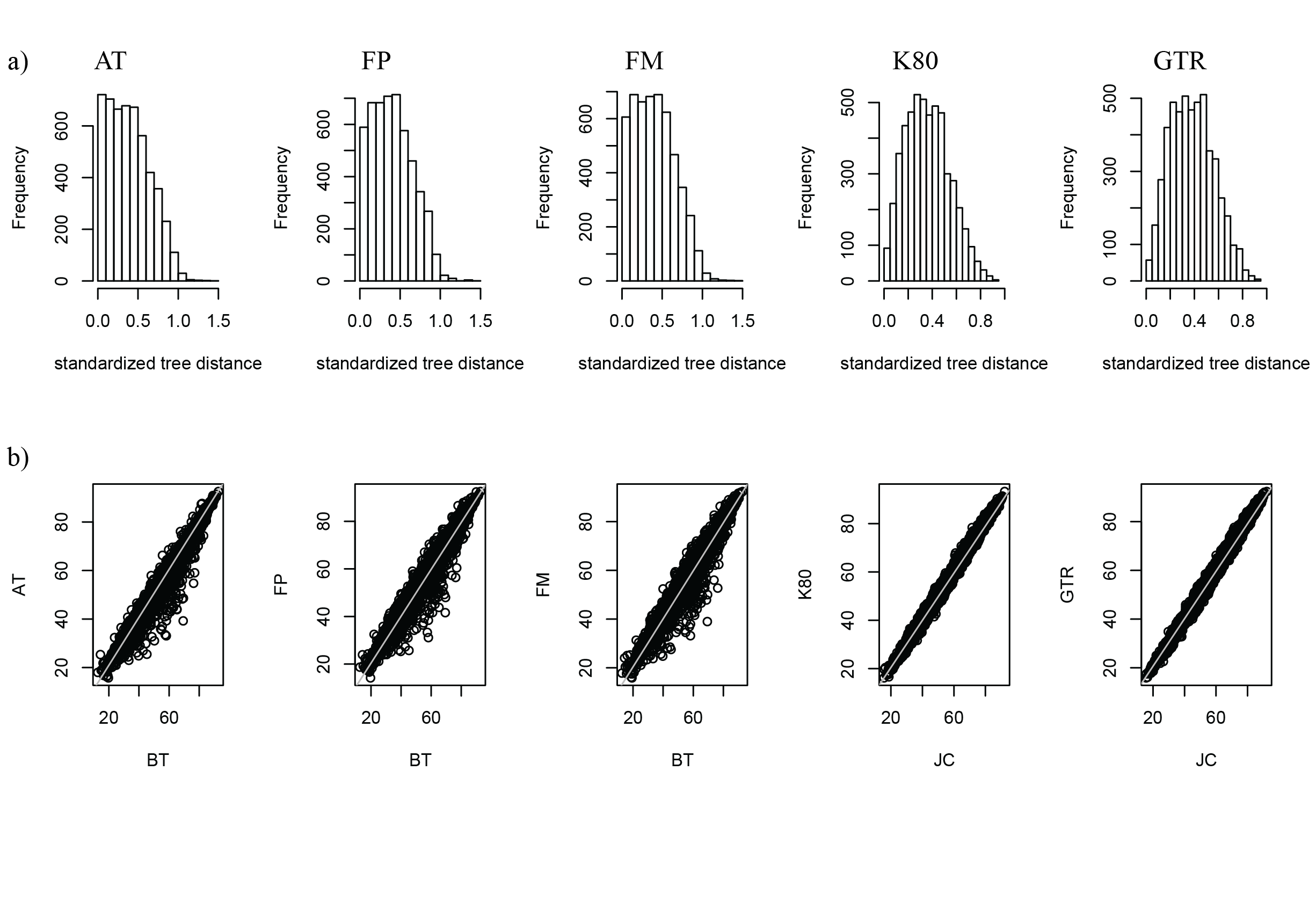
**

Figure S2. The effect of alignment and substitution model uncertainty on gene tree estimation. ML and bootstrap gene trees were estimated from the BT, AT, FP, and FM alignments. a) Histogram of the standardized tree distance across 5,162 loci. The distance between two ML gene trees is standardized by the maximum distance calculated by bootstrap gene trees. In the first three plots, the ML gene trees of the AT, FP, and FM alignments are compared with the ML gene trees of the BT alignments, and the maximum distance is calculated by the bootstrap gene trees of the BT alignments. In the last two plots, the ML gene trees estimated with the K80 and GTR models are compared with the ML gene trees estimated with the JC model, and the maximum distance is calculated by the bootstrap gene trees estimated with the JC model. b) Scatter plot of bootstrap percentages across 5,162 loci. The first three plots are the bootstrap percentages of the gene trees estimated from the AT, FP, and FM alignments against the BT alignments, and the last two plots are the bootstrap percentages of the gene trees estimated with the K80 and GTR models against the JC model.


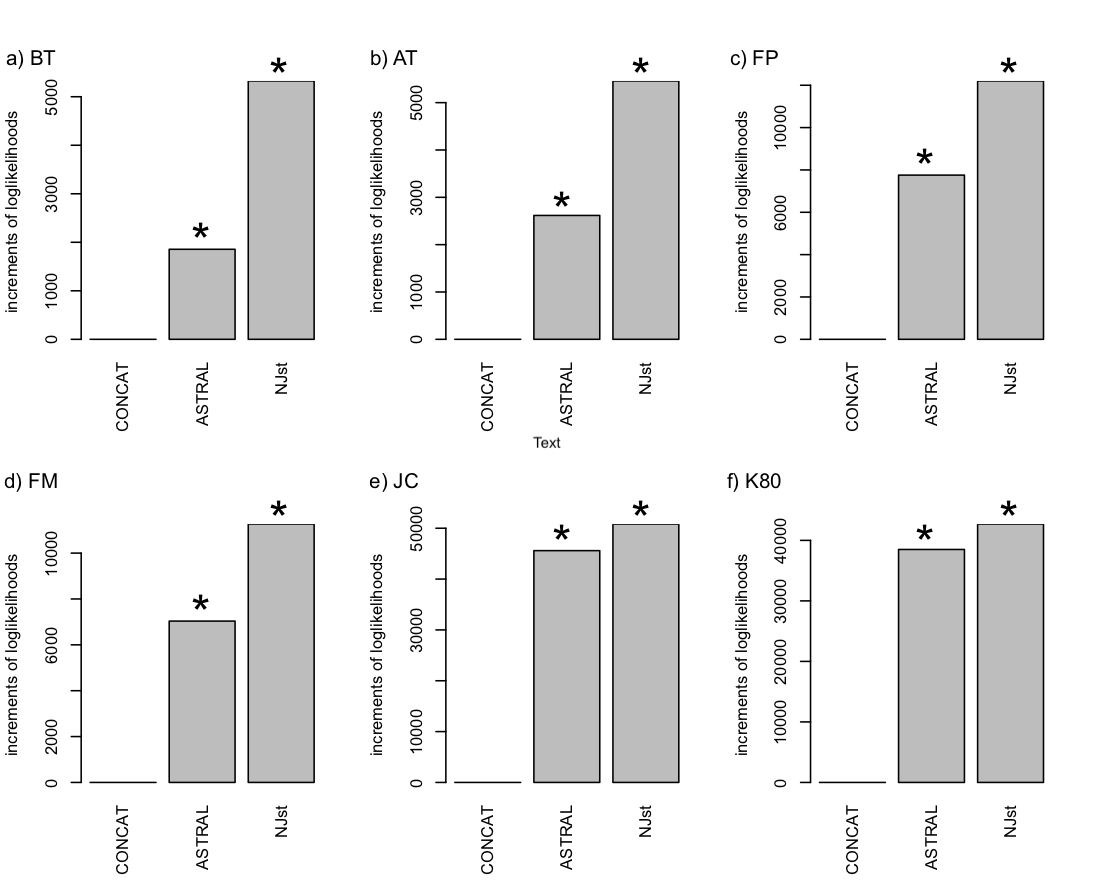


Figure S3: Significant incongruence among the estimated species trees by a likelihood ratio test. The LRT is conducted to compare the concatenation tree (the null tree) versus the ASTRAL tree (the alternative tree), and the ASTRAL tree (the null tree) versus the NJst tree (the alternative tree) for a) BT, b) AT, c) FP, d) FM alignments and e) JC, f) K80 substitution models. The log likelihoods of three trees are calculated by MP-EST, and then subtracted from the minimum of three loglikelihoods. Asterisks indicate that the test rejects the null tree and favors the alternative tree with *pvalue* < 0.01.


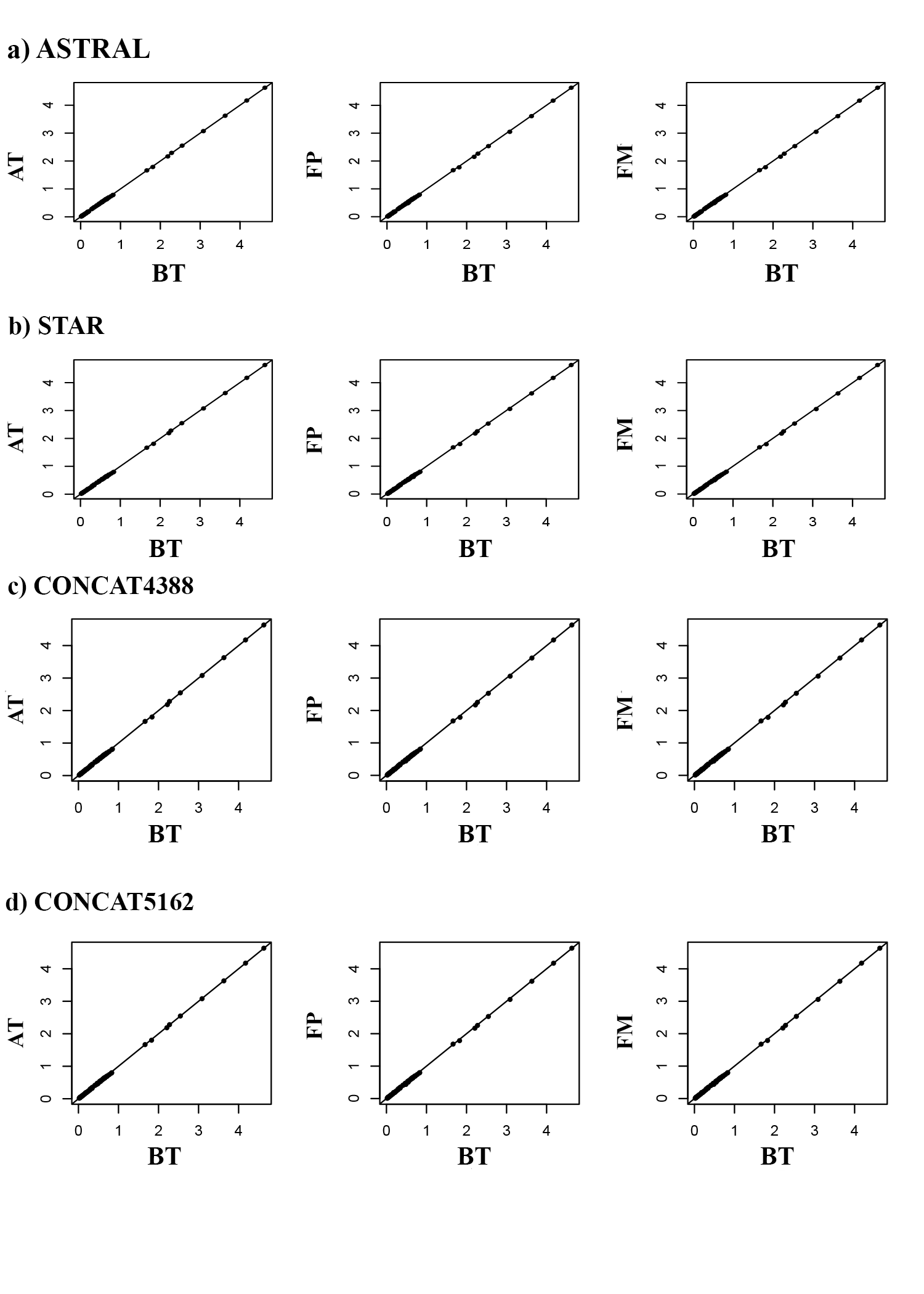


Figure S4: The effect of alignment uncertainty on divergence time estimation. The posterior means of divergence times estimated with MCMCtree for the AT, FP, and FM alignments are plotted against the posterior means of divergence times for the BT alignments for a) the ASTRAL tree, b) the STAR tree, c) the concatenation tree in Liu et al. [1], and d) the concatenation tree estimated from the FM alignments of the C12 data sets in this study.

Figure S5: The mammal time tree estimated from the FM alignments. Divergence times were estimated using MCMCtree from the FM alignments on the fixed STAR tree topology. The transparent shading indicates the Cenozoic era. The arrow and two vertical lines indicate the KPg boundary at 66 Ma and two diversification rate shifts at 54 Ma and 83 Ma estimated by the birth-death-shift model [2].  which are consistent with the estimates (54 Ma and 88 Ma) in Liu et al. [1].

References

1. Liu L, Zhang J, Rheindt FE, Lei F, Qu Y, Wang Y, Zhang Y, Sullivan C, Nie W, Wang J *et al*: **Genomic evidence reveals a radiation of placental mammals uninterrupted by the KPg boundary**. *Proc Natl Acad Sci U S A* 2017, **114**(35):E7282-E7290.

2. Stadler T: **Mammalian phylogeny reveals recent diversification rate shifts**. *Proc Natl Acad Sci U S A* 2011, **108**(15):6187-6192.
